# Idiopathic pulmonary fibrosis in 2011: key updates on guidelines and therapeutics Concluding remarks

**DOI:** 10.1186/1465-9921-14-S1-S8

**Published:** 2013-04-16

**Authors:** Ulrich Costabel

**Affiliations:** 1 Ruhrlandklinik Essen, Universitätsklinik, Tüschener Weg 40, 45239 Essen, Germany

## 

This was a very successful meeting, which brought together leading experts in the treatment and research of IPF and provided a forum for the discussion and sharing of new developments, committee guidance and clinical experience. The 2011 guidelines from the ATS/ERS/JRS/ALAT joint committee [1] provide a very good background for physicians managing patients with IPF, giving great detail in terms of the approach to diagnosing patients. Newly refined criteria were published, which gave a central role to the usefulness and implementation of high-resolution computed tomography scanning in the diagnostic algorithm.

During the meeting, there was much discussion around the daily challenges faced by physicians who have patients with IPF, which remain even after the publication of the updated guidelines. Although considered highly useful by the majority of attendees, it was generally felt that an update to the 2011 publication would be useful, to fully incorporate research outcomes that weren’t included in the existing analysis, such as the complete dataset regarding the efficacy of pirfenidone and the closing of studies of warfarin treatment, along with safety data regarding the N-acetylcysteine, azathioprine and prednisone combination therapy. It is now possible to reassess the treatment recommendations of these agents. In addition, the presentations and discussions held at the meeting identified specific issues within the diagnosis recommendations. By addressing these points, we will have an even stronger platform on which to base our patient management approaches, and will be able to provide a more confident diagnosis along with the most effective treatment options available to date. This is a very exciting time in IPF research, and we, as physicians, are driving the progress and trying to improve the outlook for patients with this difficult-to-treat disease. A key take-home message from this meeting is that each patient should be viewed as an individual case when considering the diagnostic and disease management approaches. As previously mentioned, with continuing support and enthusiasm from its attendees, the AIR Meeting should become an annual event in the calendar for respiratory physicians and researchers dedicated to advancing the treatment and management of patients with IPF.

## Competing interests

Ulrich Costabel has received consultancy fees from Actelion, Boehringer Ingelheim, Centocor, Gilead, and Intermune, and has received lecture fees from Intermune.

## Acknowledgements

The author thanks C. Trenam, I. Mandic and M. Smith of IntraMed Communications for editorial assistance in the preparation of the manuscript. Development of this article was supported by InterMune AG.

## Declarations

This article has been published as part of *Respiratory Research* Volume 14 Supplement 1, 2013:IPF in 2011 – Key updates on guidelines and therapeutics. The full contents of the supplement are available online at http://respiratory-research.com/supplements/14/S1. Publication of this supplement was supported by IntraMed Communications with funding from InterMune, AG. InterMune is the manufacturer of pirfenidone, a product mentioned in this article. The supplement originates from presentations given at the “AIR Event: Advancing IPF Research. Working together to translate IPF research into practice” held in Berlin in November 2011. The publication was proposed by IntraMed Communications and developed in consultation with the journal. All articles in the supplement have undergone the journal’s standard peer review process.
